# DNA Origami Design: A How-To Tutorial

**DOI:** 10.6028/jres.126.001

**Published:** 2021-01-08

**Authors:** Jacob M. Majikes, J. Alexander Liddle

**Affiliations:** 1 National Institute of Standards and Technology, Gaithersburg, MD 20899, USA

**Keywords:** DNA nanofabrication, DNA origami, self-assembly

## 1 Introduction

Deoxyribonucleic acid (DNA) origami is a powerful approach for fabricating nanostructures with molecular precision over length scales of approximately 100 nm. In addition, the ability to functionalize nanoscale objects with DNA, and other attachment chemistries, means that origami can be used as a molecular "breadboard" to organize heterogeneous collections of items such as biomolecules, carbon nanotubes, quantum dots, gold nanoparticles, fluorophores, etc.

On completing this tutorial, readers will have learned how to apply a basic design approach for developing DNA origami nanostructures. Since the design-property relationships of such nanostructures are still a subject of active research, we have chosen to focus on basic concepts, rather than trying to create a comprehensive guide.

## 1.1 Audience

This tutorial is designed to be used by novice designers of DNA origami systems, particularly those performing undergraduate or early graduate-level research.

## 1.2 Education or Skill Level

Readers of this tutorial should be familiar with the physical properties of B-DNA, single-stranded DNA (ssDNA), and crossover junctions. In addition, once ready to create a structure for a specific application, the designer should determine the full list of functional requirements. This list includes answers to the following questions: What should the structure do? What specific properties are critical to the system?(tm)s performance?

## 1.3 Prerequisites

The designer should have either sufficient paper for manual design (not recommended) or a design program such as cadnano [1] (all versions sufficient), nanoengineer^®^, Parabon inSēquio^®^, or equivalent.^1^1 Certain commercial items are identified in this paper in order to specify the experimental procedure adequately. Such identification does not imply recommendation or endorsement by NIST, nor does it imply that the software, materials, or equipment identified are necessarily the best available for the purpose. A registered account with three-dimensional (3D) structure prediction servers such as CanDo [2, 3] is also recommended.

## 1.4 Tools or Equipment

Equipment includes desktop or laptop computer equipment, craft supplies for macroscale models, and DNA nanotechnology computer-aided design (CAD) software.

## 1.5 Background

The sequence specificity associated with Watson-Crick-Franklin base pairing in DNA and the ability to synthesize DNA in an arbitrary sequence [4] combine with the ease of labeling other functional nanomaterials with DNA to make DNA an ideal molecular Lego^®^. It can be used to create complex nanostructures in large quantities at low cost, and it has capabilities that are complementary to top-down nanostructure fabrication methods. However, the design approach required to design and deploy this molecular assembly tool is different compared to those used for established top-down assembly techniques.

**Fig 1:**
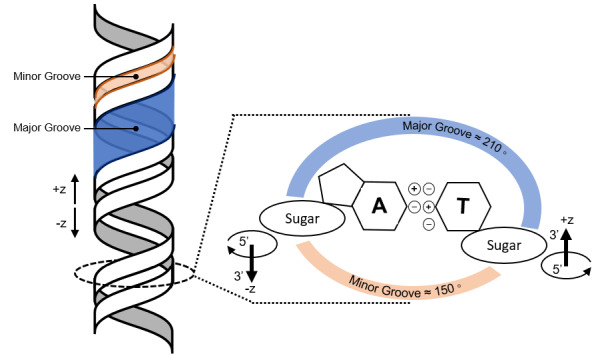
Structure of B-form dsDNA and major/minor grooves arising from base-pair geometry in which the sugar/phosphates split the head-on view of the double helix into 3.6 rad (210 °) and 2.6 rad (150 °), respectively. The phosphate groups that link the sugars of bases in the *z* axis are in and out of page in this illustration. The major and minor grooves here are not to scale.

The structure of B-form double-stranded DNA (dsDNA) is shown in Fig. 1. While the structure of DNA is a surprisingly rich field of study [5], only a few properties are relevant for programmed self-assembly [6]. These relate to the details of the lock-and-key base-pair interaction between the adenine and thymine (A-T) and guanine and cytosine (G-C) bases in DNA polymers. Due to the asymmetry of the DNA polymer and the shapes of the bases, canonical base pairs only form between antiparallel strands of DNA. DNA strands can be described as antiparallel because each strand has a chemical direction, represented by the asymmetric carbon positions (5´ and 3´) at which the phosphates are bonded to the sugar. By convention, the start of the DNA strand is called the 5´ end, because it terminates with a phosphate at the 5´ carbon position, while the final base of the strand is called the 3´ end, because it terminates with a hydroxyl group at the 3´ carbon position. Also, because the base-pair geometry is not symmetric, the helix has a minor groove and a broader major groove. If a strand were to leave the helix, *e.g.*, to make an addressable sticky end, it would be connected to the helix at the last phosphate/sugar moiety attached to a base engaged in the helix. As such, whether in crossovers or sticky ends, the choice of the *z*-axis position for a connection to the helix determines the rotation of that position around the helix.

**Fig. 2:**
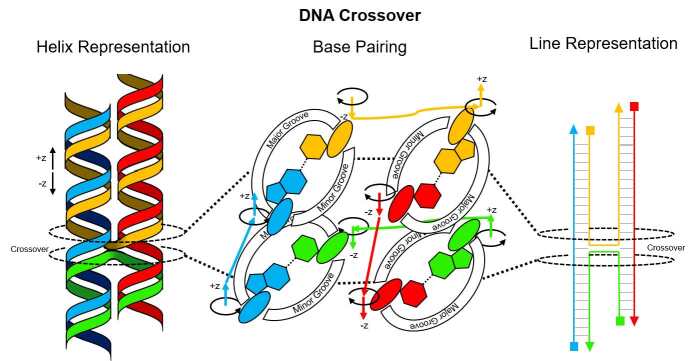
DNA crossover between helices. (Left) Helix representation. (Middle) Base pairing. (Right) Schematic line representation showing antiparallel base pairing with 5´ and 3´ ends denoted by squares and triangles, respectively.

The principal mechanism of nanostructure fabrication using DNA relies on the ability of DNA strands to cross over from one dsDNA helix to another adjacent helix, thereby connecting them. These ?ocrossovers?? can be programmed to occur at specific locations using sequence design and base pairing of ssDNA strands to create dsDNA helices. Crossovers may be formed anywhere, subject to the constraint that the phosphate backbones are aligned. Figure 2 shows the detailed helix and schematic line representations of a DNA crossover. One-dimensional (1D) self-assembly along the helix can be used to create both two-dimensional (2D) and 3D structures. The reader is directed to numerous recent reviews for structure examples [7, 8].

Side-by-side comparison of crossover representations is useful because the design process occurs using the line representation, but the rules allowing crossovers arise from physical realities visible in the helical representation. Additionally, one should note that the crossovers shown in Fig. 2 are antiparallel. That is to say, the yellow and green strands reverse their direction on being forced to exchange helices. While structures have been designed with parallel crossovers where the strands do not reverse [6, 9], they have not readily been allowed by most CAD tools and represent a minority of crossovers used.

**Fig. 3:**
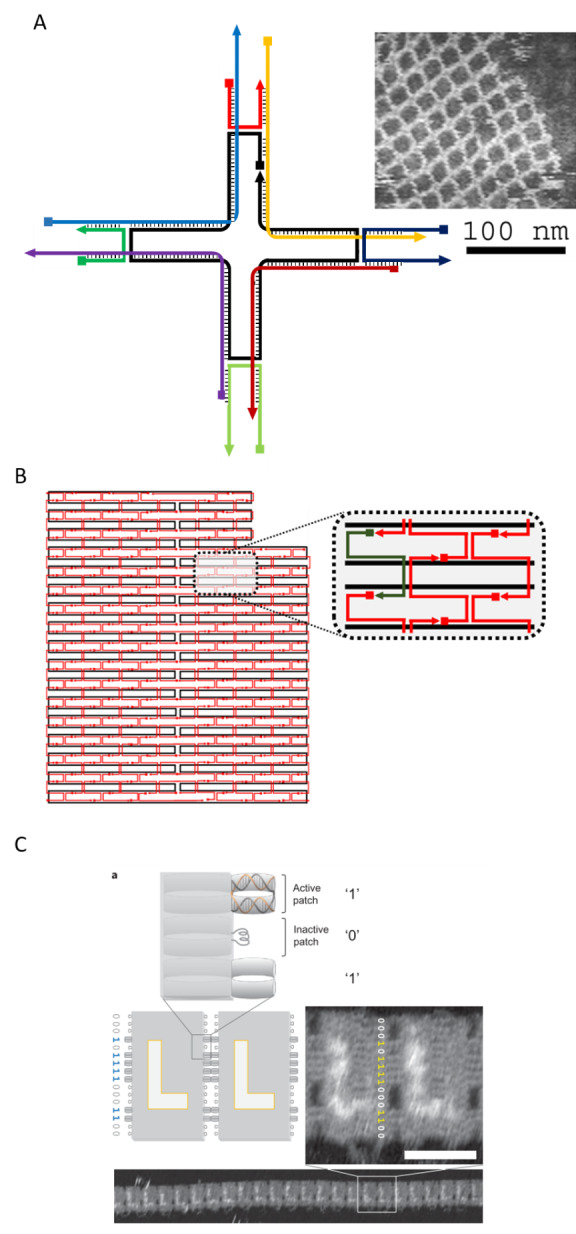
Increasing complexity in assembly. (A) DNA tile, capable of 2D polymerization, comprising four crossovers [13]. (B) Typical 2D DNA origami, containing >400 crossovers. (C) Programmed stacking between origami structures [12].

While a full review of DNA self-assembly techniques is beyond the scope of this document, we illustrate how typical structures can be built from connected crossovers in Fig. 3. DNA tiles (Fig. 3A) often require the fewest distinct DNA strands, and can polymerize in a variety of ways [6]. They are, however, notoriously sensitive to design and stoichiometry, and implementation of a tile system often requires time-consuming tuning of relative strand concentrations [6]. Developed, in part, to overcome this problem, DNA origami (Fig. 3B) comprise a long scaffold, derived from a viral genome, held together by crossovers programmed into synthetic ssDNA strands, or staples, with which it binds [10]. This approach allows the staple strands to be added in excess concentration relative to the scaffold, which drives the self-assembly towards the desired structure. DNA origami systems may be designed to assemble into larger structures *via* programmed interactions between multiple origami structures [11-13] (Fig. 3C).

While the fabrication of a new 3D structure can be readily accomplished without advanced background knowledge, the design process itself is not intuitive. Fabrication of a structure by self-assembly converts ssDNA strand sequence information into 3D topology *via* base pairing. However, the inverse operation must be performed during the design process, *i.e.*, converting a 3D structure into a linear sequence of bases. While no individual step in the design flow is unusually difficult, an organized, iterative approach significantly simplifies the process.

In attempting the design process, it is important to appreciate that current characterization capabilities limit our understanding of the design-property relationships (like yield, mechanical strength, *etc*.). This deficit in understanding increases the risk of failure for any one particular design and should be kept in mind.

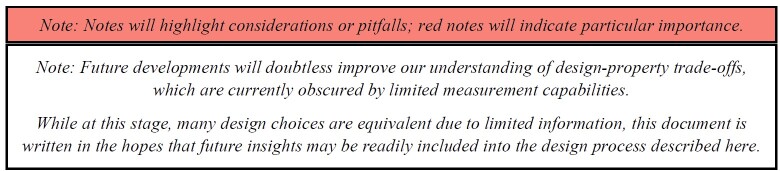



## 2 Basic Energetics, Yield, and Modeling of DNA Origami

While our understanding of the design-property relationships for many DNA nanotechnology systems is insufficient to permit development and application of general, automated design tools (although automated design tools that work within specific design constraints have been created [14-16]), there is a consensus on many underpinning concepts, described here in brief. We begin with an examination of the energetics driving the self-assembly of biological dsDNA, and we compare them with the energetics of DNA origami. Next, we discuss the yield of the assembly process in creating the desired product. Finally, we address common modeling and simulation tools used for these systems.

The energetics of classical dsDNA, *i.e.*, Watson-Crick-Franklin base pairing, are predictable based on the sequence and length of the DNA, where G-C pairings are more stable than A-T ones, resulting in a higher melting temperature (*T*_m_) for G-C-rich sequences [17]. When dsDNA forms, the hydrophobic bases are shielded from solution: Base-base interactions, called "base stacking," along the long axis of the helix are responsible for more of the favorable free energy of hybridization than the hydrogen bonding associated with A-T and G-C base pairing.

The energetics of DNA origami are at least an order of magnitude more complicated than classical dsDNA [18]. Each ssDNA strand, or staple, in an origami has multiple subsequences, allowing it to form one or more crossovers and jump between helices. Hybridization of these subsequences can behave independently for topologically distant staples or cooperatively for nearby staples, where each "correct" binding event energetically reinforces nearby correct binding events [18, 19]. This cooperativity significantly improves the quality of assembly, but it also obscures our ability to measure and understand assembly energetics. A first form of cooperativity occurs between subsequences on the same staple. When a first subsequence on an ssDNA strand binds to the scaffold, the other subsequences on that particular molecule have a dramatically increased probability of colliding with, and binding to, the scaffold. This can be equivalently described using either a unimolecular equilibrium constant including an energetic penalty for topological effects, or a bimolecular equilibrium constant with a j-factor that represents those effects [20-22]. These topological effects are the second relevant form of cooperativity. There is an unfavorable energetic contribution associated with conformational entropy loss between a free scaffold and one bound into an origami. This penalty is universal across the structure, so an individual folding event will cooperatively reduce the penalty for its neighbors. Finally, there is interstaple base stacking. If two subsequences end immediately adjacent to one another along the scaffold, then the ending hydrophobic bases, which would otherwise be exposed to water, are shielded from solution [23].

As with energetics, the yield of DNA origami is a comprehensive topic, and its full scope extends beyond this document. Yield in these systems may be defined as the number of structures that have the correct shape in microscopy, *i.e.*, imaging yield, or it may be defined through the total number of discrete staple binding defects, *i.e.*, staple yield, or it may be defined through the ratio of appropriately incorporated functional units to imaged origami, *i.e.*, functional yield. While staple yield is the most difficult to measure [24], it determines the other two, and it is the most relevant to design.

**Fig. 4:**
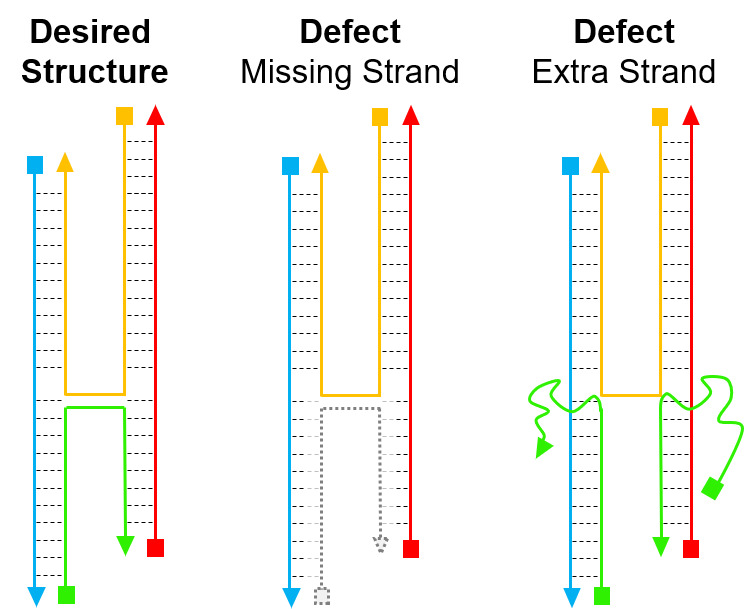
Examples of defect types for a primitive crossover.

Staple yield defects, as illustrated in Fig. 4, often take the form of missing or additional unprogrammed staples. Individual defects are often too small to detect *via* imaging, and they may occur in locations irrelevant for functional yield. In the illustrated case, the red/yellow/blue strands formed together first, but instead of correctly binding the two subsequences on a single green strand, they bound either two separate copies of the green strand or no copies of the green strand. The desired structure and the extra-strand defect have similar energetics because the same sequences/lengths of dsDNA are formed, with the only difference being a loss of translational entropy in the second copy. This loss is 3/2R, where R is the gas constant, and *T* is the temperature. This malformed structure would then have two unsatisfied subsequences floating in solution, which could potentially further polymerize. Generally speaking, these defects are avoided through careful tuning of the relative concentrations of each strand, particularly for tile-based systems [6], and through inherent cooperativity, particularly for origami [19].

Origami also has a natural stoichiometric control in which all staples bind to a single scaffold, *allowing the staples to be added in excess*, and preventing missing-strand defects. For other systems, increasing the staple concentration would dramatically increase extra-strand defects. However, the first form of cooperativity, described above as the transition from bimolecular to unimolecular binding, favors the first staple molecule to bind to a scaffold, preventing extra-strand defects even at high staple excesses. This is critical, because using high excesses of staples allows one to drive the system away from missing-strand defects. In short, origami's natural stoichiometric control allows for dramatically higher yield of the desired structure for significantly less experimental effort. We recommend the following papers for those interested in more depth on this topic [18, 25-28].

A related consideration is the synthesis yield of synthetic ssDNA. Phosphoramidite DNA synthesis adds each base in a sequence with >99% efficiency. However, the probability of synthesizing the correct sequence in its entirety is this efficiency raised to the power of the number of bases. It is not uncommon for a fraction of synthesized staple strands to be missing bases. If these missing bases occur on only one subsequence, it can guarantee an extra-strand defect. The natural stoichiometric control in origami reduces the likelihood of this, because a fully correct strand may bind in the second extra-strand position, where cooperative energetic effects will favor it to exchange and displace the strand with the incorrect sequence.

While DNA origami assembly has been modeled and simulated, there are relatively few tools accessible that do not require a significant investment to implement. However, simpler tools are available that provide some useful insights. Web-based thermodynamics calculators predict classic DNA hybridization energetics and predict unwanted hairpin formation [29-31], although these tools can typically only manage single hybridization subsequences. CanDo is a finite element analysis tool, which treats the dsDNA helices as static cylinders that can be torqued as they are welded together by crossovers and subject to thermal energy [32]. Finally, tools for molecular dynamics (MD), coarse graining, and multiscale modeling exist. MD simulates behavior by treating atoms and bonds as beads and springs, respectively, with appropriate terms for electrostatics, *etc*. [33-36]. MD is generally more accurate than coarse graining, which treats entire bases or chemical subunits of bases as a single bead [37], but consumes so much more computational power that it often cannot simulate events that occur at a timescale longer than nanoseconds. Multiscale modeling is a technique by which different levels of coarse graining and MD are simulated and interchanged [38].

## 3 Common Terms

Scaffold: Long DNA strand that is bound together to form an origami. It is often circular, ssDNA, and sourced from a virus, although this is not always the case [39].
Scaffold routing: Raster pattern through which the scaffold travels back and forth through the structure.
Crossover: Position where DNA helices exchange ssDNA strands, linking them.
Staple: Short oligomer ssDNA sequences, usually synthetic, forcing the scaffold to route into a desired shape.
Staple subsequence: Contiguous subset of the staple sequence that binds a contiguous sequence along the scaffold. Subsequences are separated by crossovers.
Staple motif: Pattern of staple subsequence divisions that reoccurs throughout a structure, *e.g.*, the 8 base-pair (bp) subsequence - 16 bp subsequence - 8 bp subsequence commonly used in 2D origami such as the Rothemund Tall Rectangle [10].
Fold: The change in topology of the scaffold when a crossover, or 1/2 crossover, forms.
Sticky end: A short subsequence left intentionally unbound at a specific position in a structure that will later be used to bind the structure and other nanoscale objects, including nanoparticles, biomolecules, and other structures.

## 4 Instructions

Design methodologies are inherently unique because of system constraints. Due to the nature of DNA synthesis, and specifically the minimum amount of material that it is feasible to synthesize or purchase, the time and money needed to produce a prototype structure are approximately the same as those required for large-scale purchases. This means that the number of practicable design iterations is limited by the expense of the design-prototype-test cycle. This tutorial attempts to apply basic engineering design theory to minimize the number of design cycles.

**Fig. 5:**
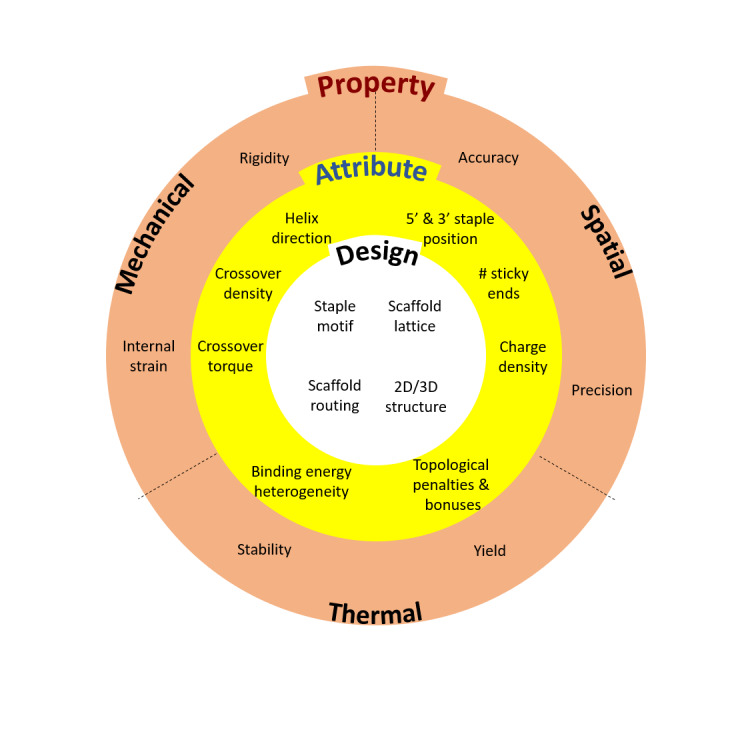
Examples of properties of interest, the attributes of a structure that determine them, and the relatively few design choices that determine those attributes in a nonorthogonal fashion.

As noted above, our ability to reduce the number of design cycles is hampered by our ignorance of design-property relationships, which is, in turn, exacerbated by the limited characterization capabilities currently available. To complicate matters further, the design space for DNA nanofabrication is large. In a very real sense, our ignorance is a meaningful constraint; without knowledge of the design-property relationships linking the items in Fig. 5, it is common for even experienced practitioners to get trapped in an infinite design-iteration loop, searching for a "perfect" structure, which may or may not be physically realizable. Additionally, given the limited number of design parameters that can be varied, any attempt to optimize one property is highly likely to dramatically change the others.

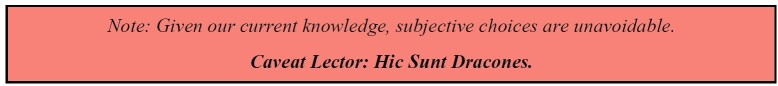



### 4.1 Predesign

As with almost any complex endeavor, the importance of predesign or preproduction cannot be overstated. Given the relative immaturity of both the field of DNA nanotechnology and the nascent CAD tool market created to serve it, the actual design process can be highly unintuitive. Beginning design without a meaningful list of specifications invites significant loss of time.

Defining Functional Requirements:

a. List all physical features relevant to the purpose/application, such as level of control needed over distance between specific functional components, overall footprint, accessibility of components to solution, chirality, stimuli response, dynamic motion, inter-structure interactions, *etc*.
b. Determine how success in creating the desired structure will be determined.
  b.I. During development: for example, transmission electron microscope (TEM), atomic force microscopy (AFM), gel electrophoresis.
  b.II. During use: for example, pharmaceutical activity, total number of actuation cycles before failure.
c. Order these physical features by their impact on the probability of success in application.
  c.I. Combine the above information into a single set of specifications.
  c.II. Add fabrication constraints, *e.g.*, monetary constraints on number of prototyping cycles.

Two constraints that pertain to the majority of origami are the length and, commonly, the circularity of their scaffolds, although both can be circumvented through application of production and enzymatic methods [40]. Practically, this means that most structures are limited to a maximum of 7249 bases, corresponding to a dsDNA length of 2389 nm or a surface area of approximately 5000 nm^2^ (6000 nm^2^ for 2D structures after relaxation and expansion).

The ubiquity of the most common scaffold, the M13mp18 genome, is in part historical and in part practical. The practical considerations are as follows. The M13 bacteriophage infects the bacterium *Escherichia coli* (*E. coli*), both of which have been researched exhaustively. The M13 bacteriophage is well situated in inherent trade-offs among the type of virus (ssDNA, dsDNA, ssRNA, dsRNA), the length of the viral genome, and the mutation rate [41]. While the M13 bacteriophage packages its genome as ssDNA, which is highly desirable for origami, its packaging accommodates varying lengths of ssDNA without significant mutation concerns. The cylindrical viral capsid consists of a handful of proteins at the caps and a repeating coat protein along the cylinder wall. For the M13 phage, changing the amount of ssDNA packaged can be accommodated with a change in the number of sidewall proteins and the concomitant length of the viral capsid cylinder. The use of the mp18 sequence specifically, rather than another version of M13, is largely historical, since it was used in Paul Rothemund's initial work on DNA origami [10]. While longer and shorter scaffolds have been developed and employed [42, 43], the success and utility of the original have limited the drive to explore this design variable in detail.

### 4.2 Macroscale Prototyping

Because true nanoscale prototypes are infeasibly expensive in both time and money, macroscale prototyping is critical to efficient design. Mockups can be made with pen and paper, dowel rods, magnetic toys, *etc*. Note: Colored magnetic bars are particularly useful for this purpose.

**Fig. 6:**
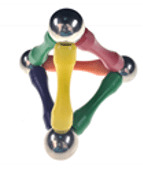
Tetrahedron mockup.

Creating mockups is largely iterative, as final and initial designs are rarely identical. If at any point it is likely that the specifications and constraints cannot be satisfied for a given structure, reiterate from the most relevant step.

a. Build a mockup (Fig. 6).

Attributes of effective mockups include the following:

● It is easy to label and relabel subsections.
● They are easy to assemble/disassemble.
● They have rotatable joints for easier flattening.

b. Estimate whether specification is feasible.

**Fig. 7:**
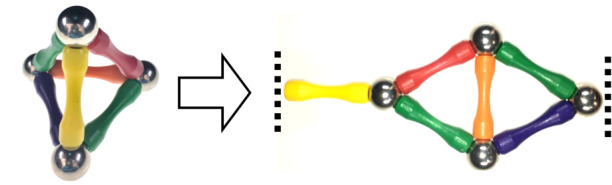
(Left) 3D mockup. (Right) Flattened mockup.

For example, for each edge/surface of a structure, determine:

● How many helices will run through this edge/surface?
● Given the number of helices and relative proportions, can the physical specifications and the constraint on dsDNA length be met simultaneously?

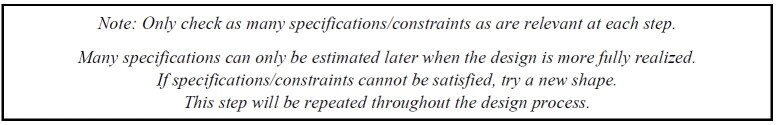



c. "Flatten" the mockup (Fig. 7).

**Fig. 8:**
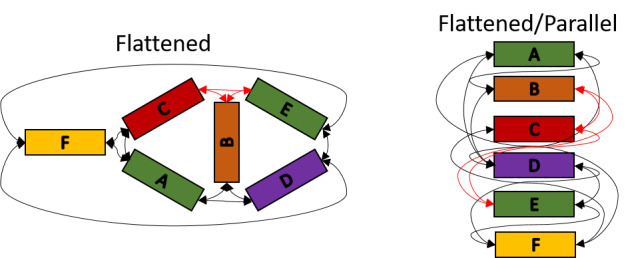
(Left) Annotated, flattened mockup schematic including topological links. (Right) Flattened mockup schematic after parallelization of helices.

**Fig. 10:**
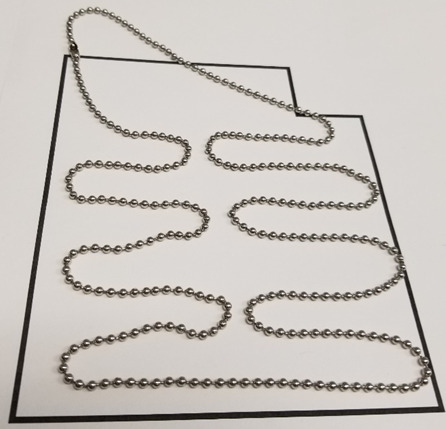
Physical mockup of scaffold routing using a beaded chain.

Both structure design by hand and most available CAD tools route the scaffold in 2D and force all helices to appear as if they are parallel. It is therefore important to be able to flatten the model to visualize the relationship between the design and the CAD representation.

d. Identify and label the subsections and their interconnections (Fig. 8). For an additional example of the importance of this concept, see CAD drawing in Fig. 12.

e. Select the helix arrangement, also called a scaffold lattice (Fig. 9), and mock up a routing pattern (Fig. 10).

**Fig. 9:** Scaffold lattice examples for honeycomb (top) and square (bottom) lattices. Dotted circles indicate potential locations for other helices, and full cylinders indicate helices in the design. **Table:** 

	**2D**	**3D**
***Scaffold Lattice*** ***or*** ***Helix Arrangement***	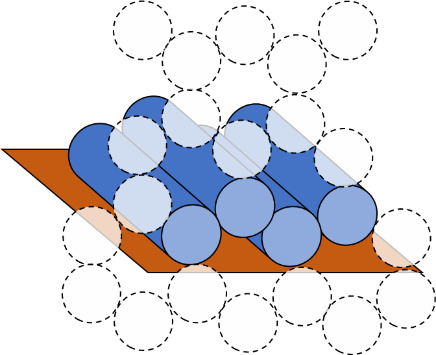 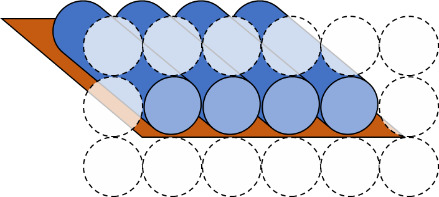	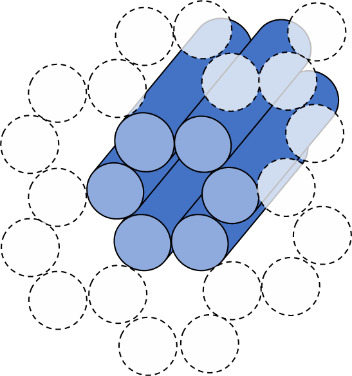 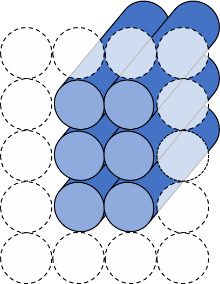

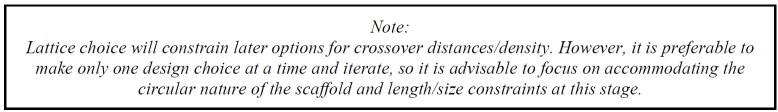

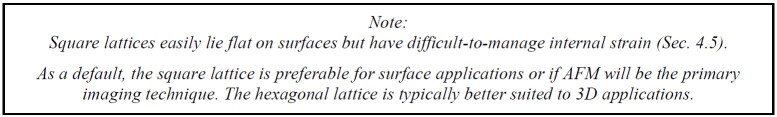





f. Using the mockup routing pattern, draw a full routing pattern (Fig. 11).

**Fig. 11:** Example routing patterns for (left) 2D square lattice structures and (right) 3D hexagonal lattice structures. **Table:** 

	**2D**	**3D**	
***Scaffold Routing***	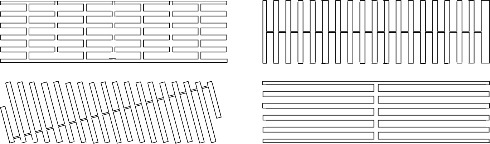		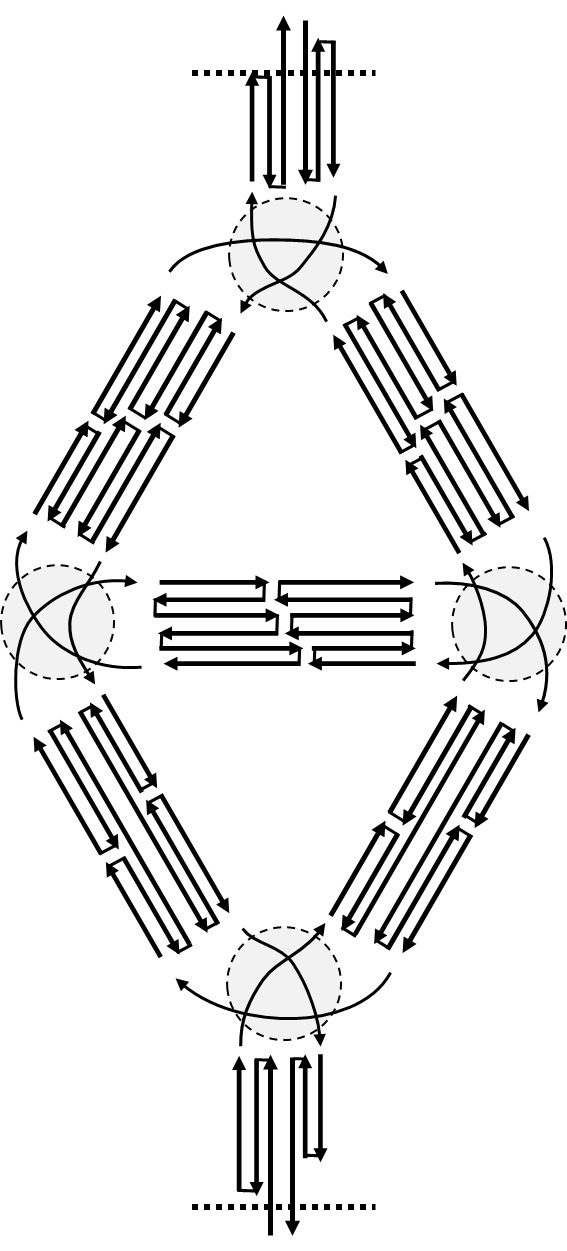

g. Repeat step -As the rough design is now more complete, confirm that specifications can be met:

● Is it physically possible to perform a valid scaffold routing given that the scaffold is circular?
● Are length/size specifications still feasible?

### 4.3 Computer-Aided Design (CAD)

For convenience, the cadnano2.0 [1] (cadnano.org/ & github.com/douglaslab/cadnano2) design tool will be used as an example in this section. Other CAD tools, such as scadnano (scadnano.org/), SAMSON-adenita toolkit [44] (www.samson-connect.net/elements.html), vHelix [45] (vhelix.net/), and Daedalus [46] (daedalus-dna-origami.org/), are readily available and may have their own advantages. As many of these tools are open-source tools, we used a GIT tool, GitHub desktop, to ensure operation of the most recent version and to minimize installation problems.

Figure 12 shows two example designs and CAD screenshots. The visual complexity associated with the routed scaffold and 200+ interconnected staples can make CAD modification difficult. Because of this, emphasis is placed on carefully planning both the design and the CAD file to make both visualization and modification less cumbersome. Some CAD tools allow for rotation of helices within a design (Fig. 12), which can reduce some, but not all, visual clutter.

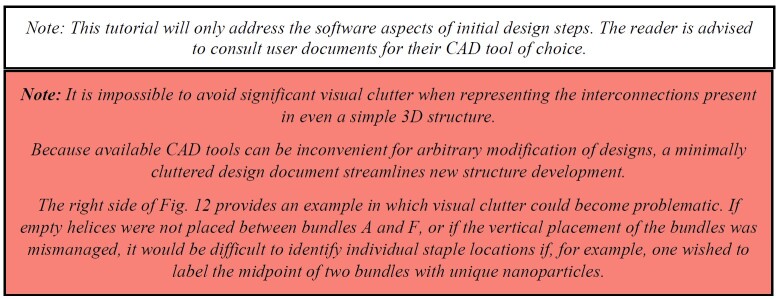



**Fig. 12:** Examples of origami design and CAD screenshot. (Left) Notched rectangle structure. (Left-Top) Graphic representation of design. (Left-Bottom) Screenshot of CAD screenshot. (Right) Tetrahedron structure. (Right-Middle) cadnano CAD screenshot. (Right-Bottom) scadnano CAD screenshot. **Table:** 

*Notched Rectangle (2D)*	*Tetrahedron (3D)*
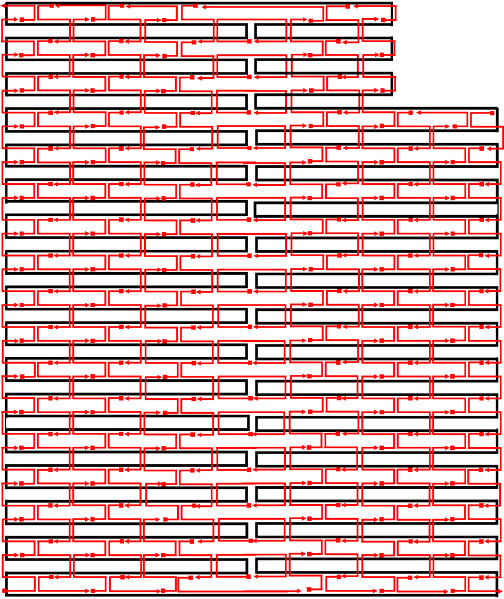 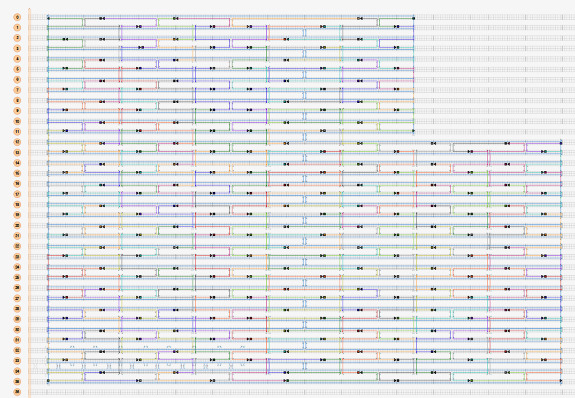	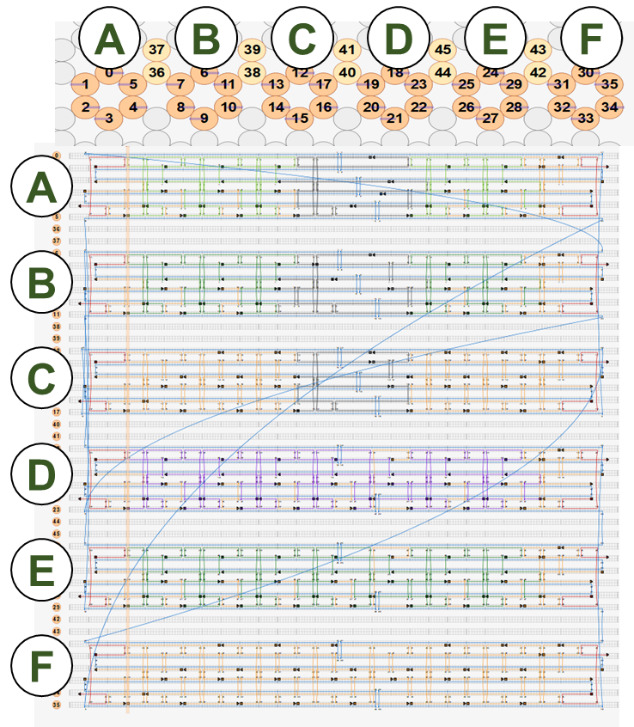 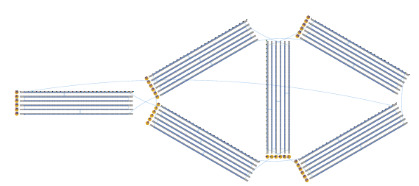

h. Define the workspace (Fig. 13A-D).

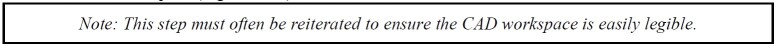

h.I. Select helix type (Fig. 13A).
h.II. Define helices (Fig. 13B-C). For cadnano, the left window looks down the *z* axis of the helices, while the right window has the *z* axis running from left to right.
h.III. When helices on the left are clicked, an "empty" helix on the right will appear.
- In cadnano2.0, the helices cannot be reordered. It is advisable to add "empty" helices between substructure units to make them visibly distinct from one another, *e.g.*, A-F in the tetrahedron in Fig. 12.
h.IV. Clicking again, holding, and dragging across the helices on the left will automatically generate a scaffold routing between all the helices over which the mouse was dragged over (Fig 13D).

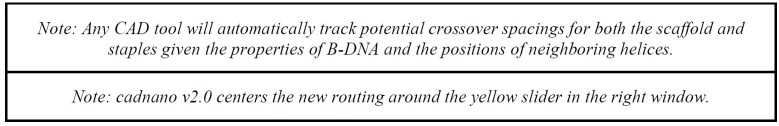

**Fig. 13:** (A-D) CAD images corresponding to step h. (A) Lattice selection determining staking of helices when looking down the z axis. (B) Selection of helices to populate into CAD file. (C) Populated helices for a six-helix bundle. (D) Automatic scaffold routing created by dragging mouse across helices in C. **Table:** 

**A**	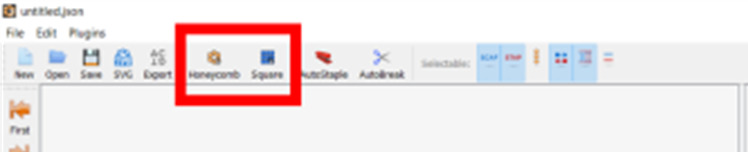
**B**	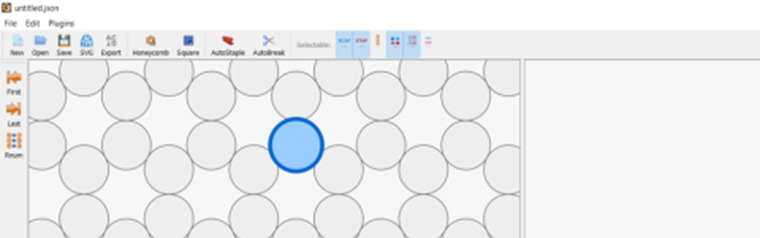
**C**	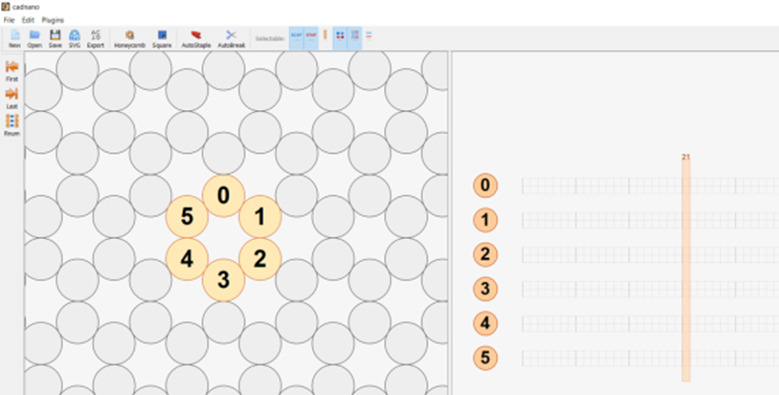
**D**	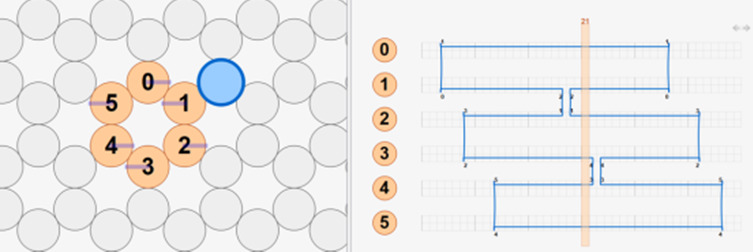

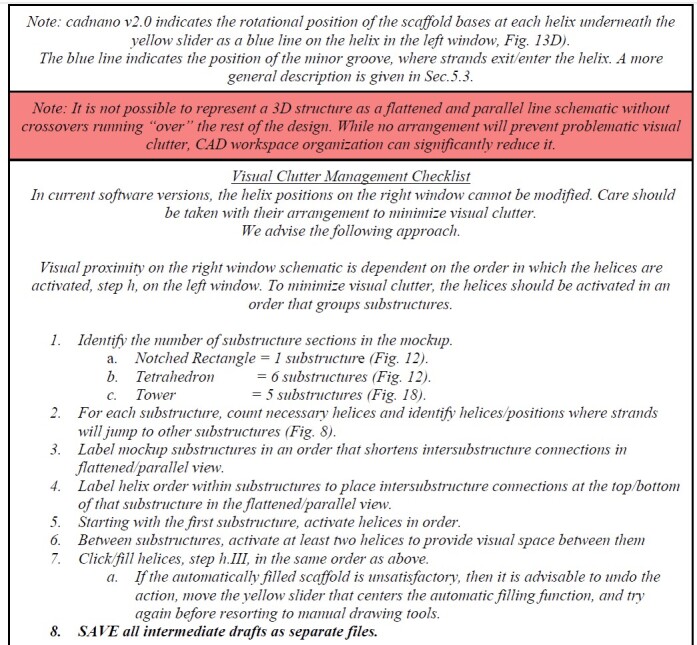

i. Test staple motifs on a small section of the scaffold routing pattern. For examples, see Fig. 14.
i.I. The goal, at this step, is to confirm that a valid staple motif exists for the chosen scaffold routing that can meet the design specifications and constraints.
i.II. Staples may be drawn manually, or they may be edited after generation *via* cadnano's "autostaple" function.
  ● Consider the following criteria when evaluating staple motifs and design specifications.
    - Consider the staple fit over the scaffold "seams," because positions where the scaffold crosses over between helices are typically the longest distance folds, and the most susceptible to defect formation:
      o Fewer than 5-8 bases of connection may not have desired stability.
    - Consider the distance between, and number of, crossovers:
      o More crossovers => stiffer structure (along crossover axis).
      o More crossovers => more internal strain (addressed in *initial testing and fine tuning*).
    - Consider the 5´ and 3´ (start and stop) position locations that are natural locations to extend sticky ends and create functional binding sites (addressed in *additional conceptual tools* section).

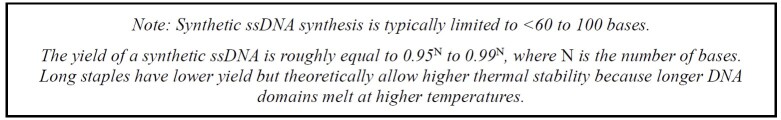

**Fig. 14:**
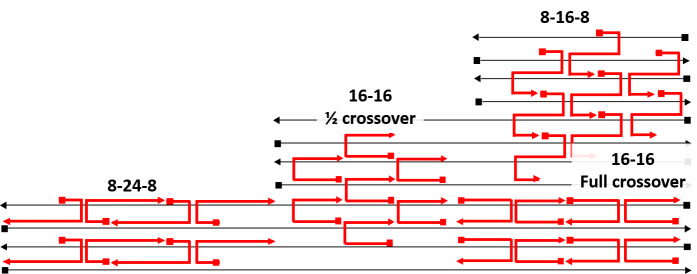
Example staple motifs on the square 2D lattice, with tentative naming convention based on the number of bases in each subsequence with differentiation for full and half crossovers where necessary.

j. For testing, we advise the following steps (Fig. 15).

j.I. Confirm the autostaple pattern has satisfactory crossover options; if not, then delete and recenter the scaffold routing (Fig. 15A).
j.II. Treat the scaffold seam regions separately from the rest of the structure.
j.III. Identify a staple motif rule, *e.g.*, one 8 base subsequence, one 16 base subsequence, and one 8 base subsequence, preferably with a nucleating subsequence [47].

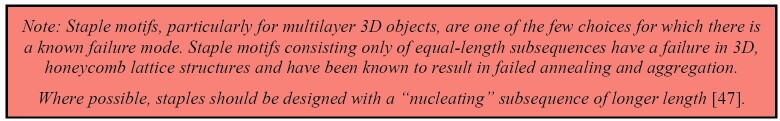

k. Save a copy of the scaffold routing empty of staples (Fig. 16).

l. Attempt to tile selected motif over a small area. Write down or otherwise confirm the tentative pattern of the staple motif.

m. Estimate feasibility of meeting specifications in step b.

n. Populate the routing pattern with the tentative staple motif choice from step j.III. An example of this is shown in Fig. 12.

o. Save the completed file under a new file name.

**Fig. 15:** Example tests for staple motifs. (A) Initial scaffold routing. (B) Staple crossover pattern generated by the autostaple function. (C) Incremental division of the staple crossover pattern into individual staples of a length that can be ordered for custom synthesis. **Table:** 

**A**	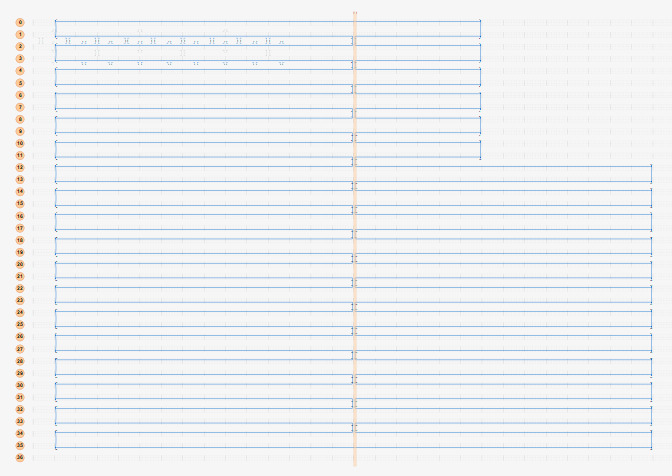	**C**	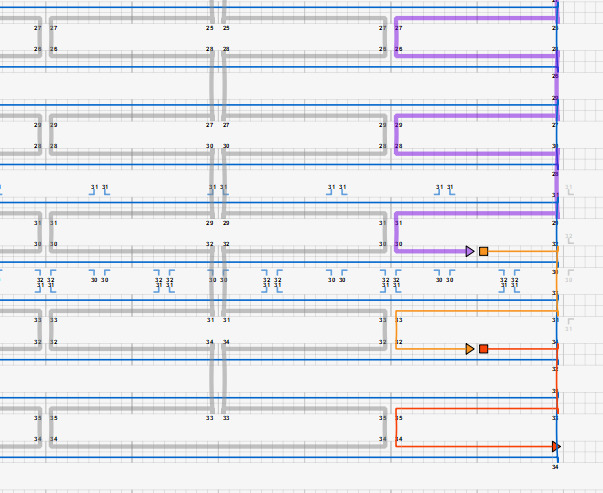 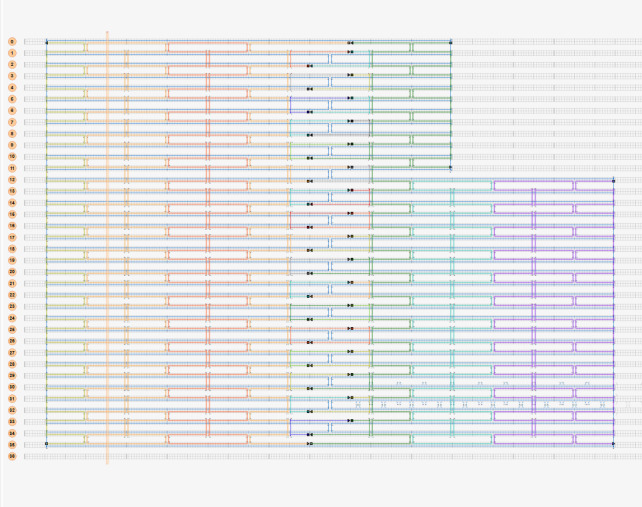
B	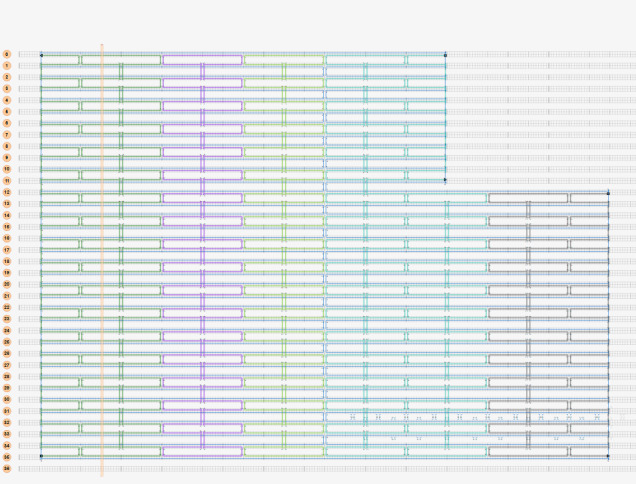

**Fig. 16:** Example routing maps and final staple screenshots matching Fig. 12. **Table:** 

*Notched Rectangle (2D)*		*Tetrahedron (3D)*
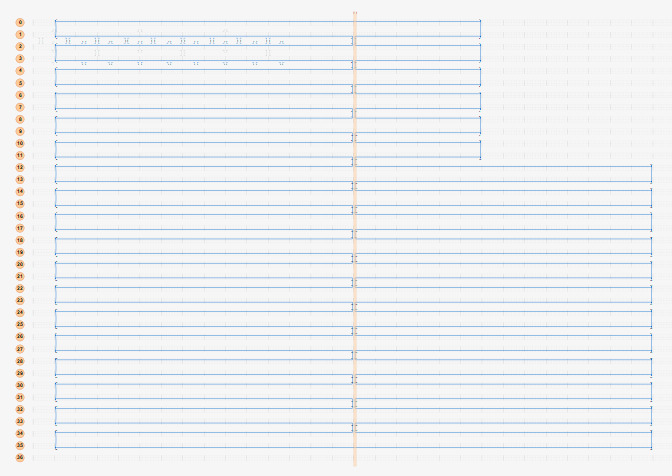 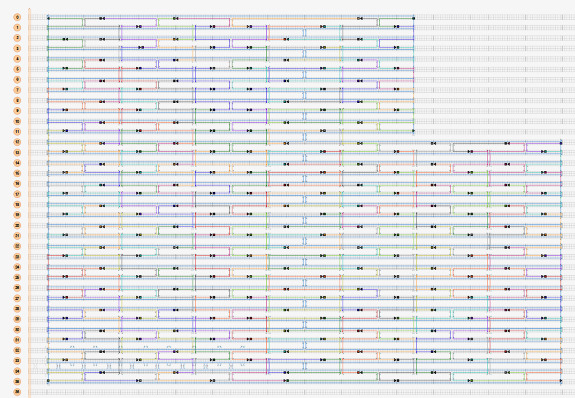	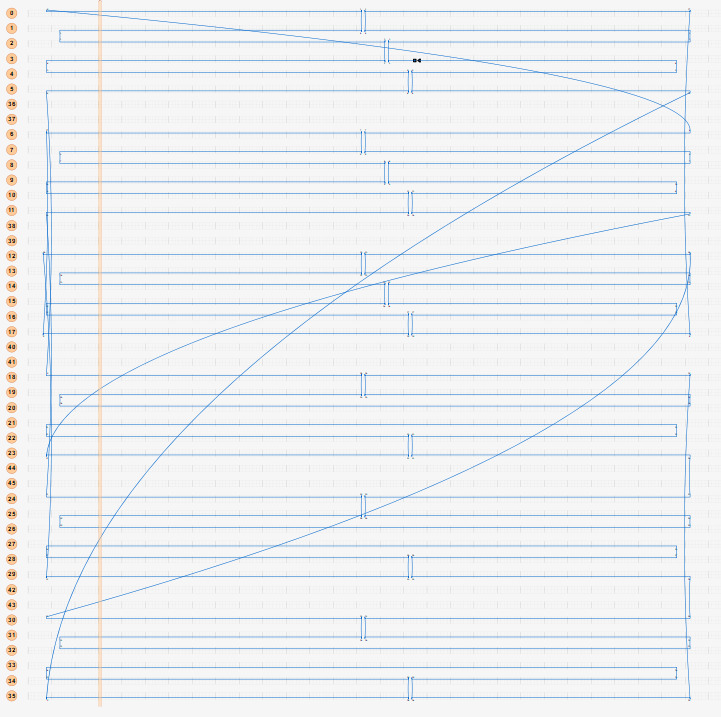 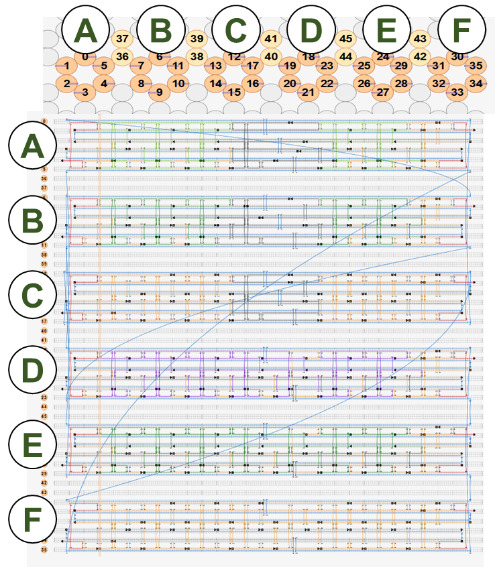

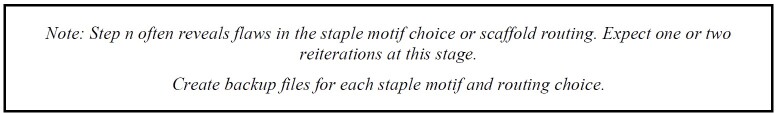



### 4.4 Initial Structure Prediction

Numerous tools exist for evaluation of structures. While we use CanDo (https://cando-dna-origami.org/), other tools, such as circleMap (https://nanohub.org/resources/cadnanovis) and University of Illinois at Urbana-Champaign (UIUC) multiscale (http://bionano.physics.illinois.edu/origami-structure), also exist. Physics-based simulations such as UIUC multiscale or oxDNA [37] may provide more nuanced predictions at the cost of time and expertise to initiate. An advisable first step is to test new structures with a finite element analysis tool such as CanDo. Finite element analysis treats each helix as an independent cylinder and the crossovers as welds with nominally realistic levels of torque and strain given the angle of the crossover and physical properties of DNA. Finite element analysis provides a preliminary confirmation of the 3D structure, and it is preferable to performing an MD, coarse-grained, or multiscale modeling simulation on the structure, which could potentially waste significant computational resources.

**Fig. 17:**
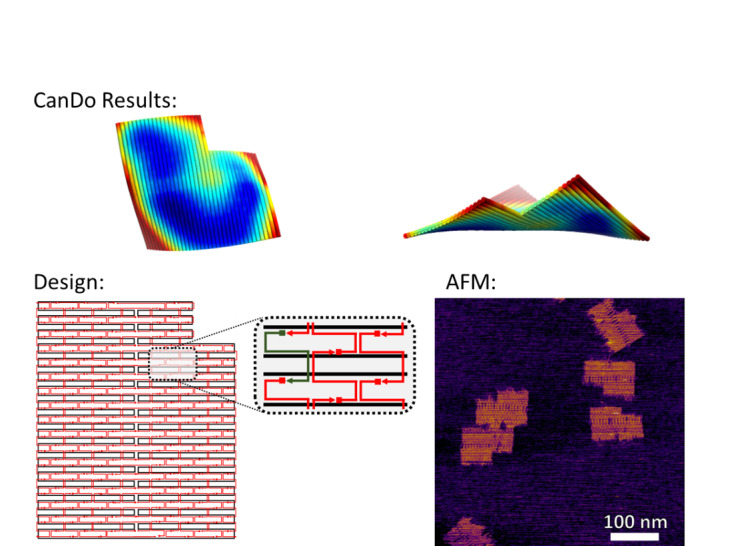
Notched rectangle design, CanDo result, and AFM image.

**Fig. 18:**
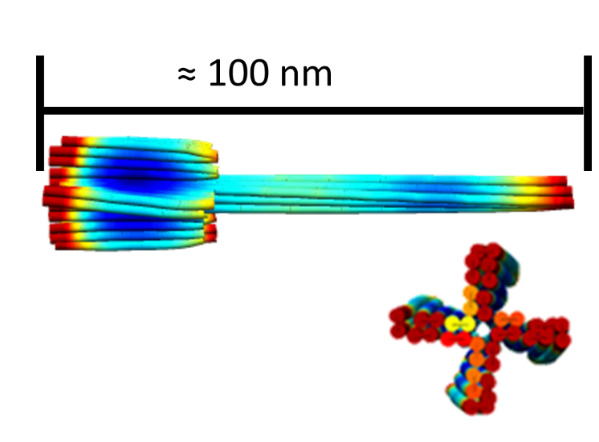
Tower origami CanDo result.

If curvature of the structure is irrelevant to the design specifications, then the need for testing and fine tuning may be limited. Figure 17 shows the CanDo simulations on the notched rectangle and an AFM image thereof. The curvature in this CanDo simulation is a reasonable representation of the effects of internal strain and torque on 2D origami structures [48]. A 3D tower structure on the square lattice is shown in Fig. 18.

For CanDo, red indicates a section of the structure that is freer to move, while blue indicates a static section. CAD modification at this stage is highly dependent on design specifications. One common modification, strain compensation for square lattice structures, will be addressed in the next section.

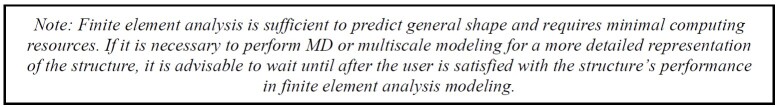



### 4.5 Crossovers and Internal Strain

When addressing internal stress, strain, and torque of DNA origami, it is doubly important to know in advance which functional requirements one is pursuing. While the interplay among design, stress, strain, and torque is relatively easy to model, they are often difficult to visualize and implement. As a result, it is easy to waste significant time and effort modifying these factors to no positive effect.

**Fig. 19:**
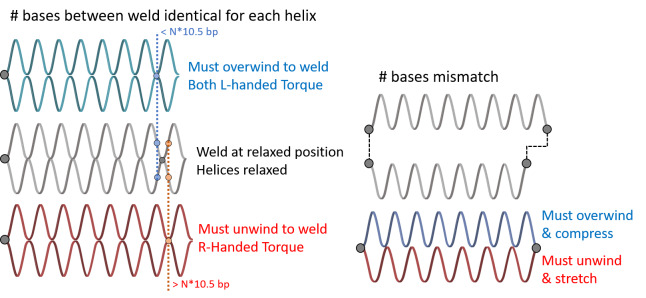
Spring analogy for over- and underwinding of DNA when helices are connected by crossovers. L-handed and R-handed indicate left-handed and right-handed, respectively.

Internal strain and torque in DNA origami are well described by the analogy of welding together springs, as illustrated in Fig. 19, where the metal of the spring represents the minor groove. In this analogy, there are two springs that must be joined at two positions to create a larger structure. Both springs have the same helicity and period. After the first weld, the springs are aligned, and there is a visible periodicity where the metal of each spring touches. At these positions, one could apply a second weld with no tension or torque. However, if one wished to weld at two positions of equal distance to the first weld, but a little before or after this period, one would have to tighten or loosen the spring to bring those points into line. Similarly, if one wished to weld the springs at slightly different lengths, one would simultaneously have to compress and tighten one spring while stretching and unwinding the other.

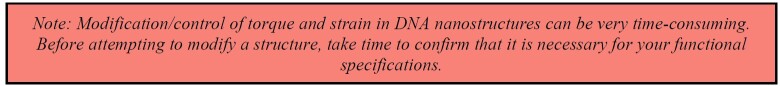



The spring analogy is imperfect, DNA cannot be treated as continuous, and crossovers can only occur between integer numbers of bases. This can be problematic because the dsDNA helix has 10.5 bases per full turn. The number of bases between potentially relaxed crossover positions depends on the angle between helices being welded (Fig. 23), which can confuse the process of choosing the number of bases between crossovers for a particular design. Binding numerous helices at a variety of angles is much more complex than simply welding two springs.

**Fig. 20:** (Left) Cylinder representation of dsDNA between crossovers. (Middle) Resulting torque within a structure if only the center purple helix/cylinder from the left side is overwound or underwound. (Right) Schematic of strain in DNA crossovers. Torque and strain will occur simultaneously in most systems as the sum of small contributions from all crossovers. They are depicted here as independent for illustrative purposes only. As in Fig. 19, red indicates overwound helices, and blue indicates underwound helices. **Table:** 

**Relaxed/Example**	**Torque**	**Strain**
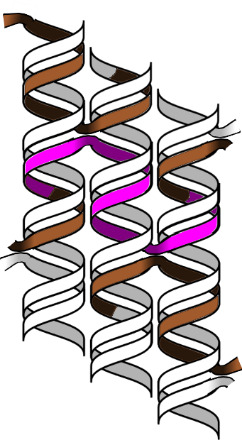 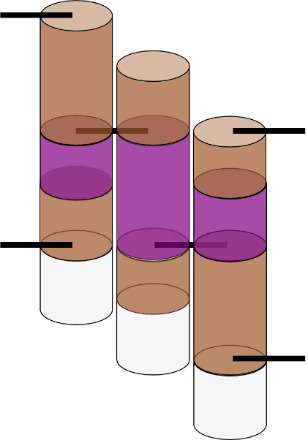	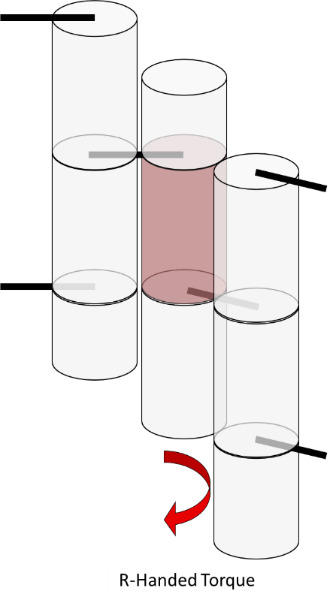 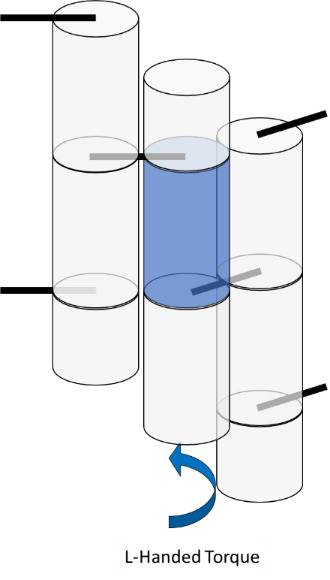	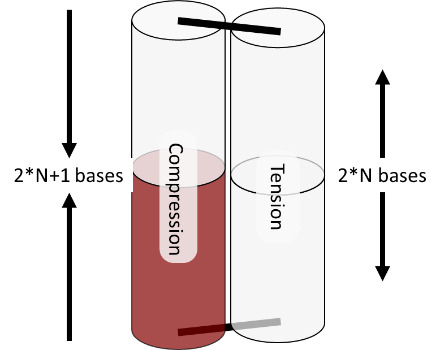 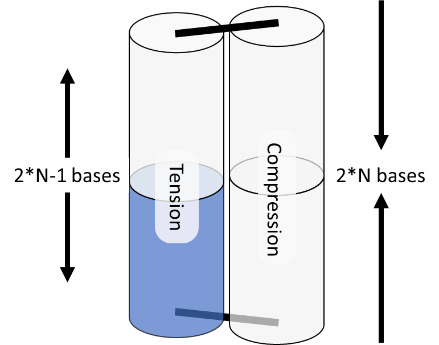

**Fig. 21:**
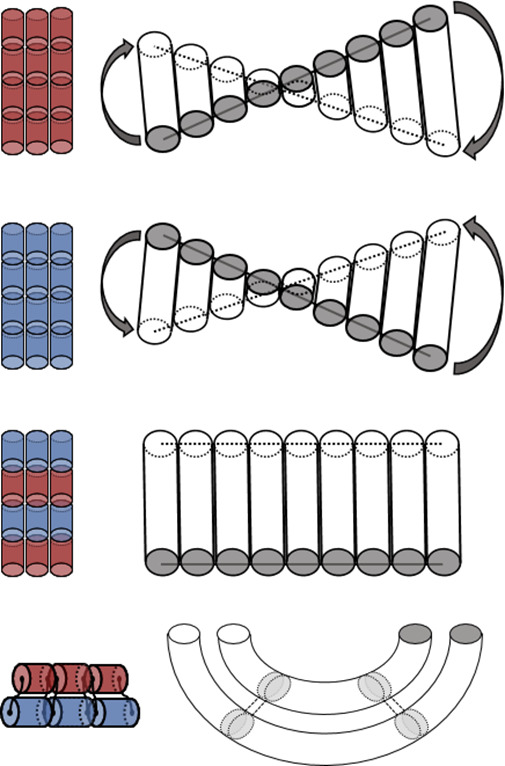
(Left) Arrangement of strained/torqued helices. (Right) Approximate effect on overall structure conformation of that strain/torque arrangement. As in Fig. 19, red indicates overwound helices, and blue indicates underwound helices.

The torques and compressive/tensile forces may be controlled by varying the number of bases between crossovers (in cadnano v2.0, by using the skip and insert tools). The effects of these approaches are shown schematically in Fig. 20 and Fig. 21. Additionally, Fig. 22 uses a full origami CAD example.

The constraints on staple length dictated by yield in ssDNA synthesis and specifications for flexibility (requiring more/fewer crossovers) are important to consider because torque and strain are controlled *via* changing crossover spacing with skips and inserts. The default spacing is a set number of bases in the software for each lattice/angle between helices. This number must be an integer, but the relaxed number of bases for that angle is often not an integer. A skip, when placed between crossovers, informs the software to have one base fewer than the default between those crossovers. An insert informs the software to have one base more than the default.

**Fig. 22:** CanDo example of DNA skips and strain compensation on a square lattice for a planar origami. The top row shows the handedness of the torque as determined by the presence and location of skips. Red indicates right-handed torque helices, and blue indicates left-handed torque in the twist schematic. The average twist values are indicated for each of the four designs. As noted in the text, the number of base pairs per turn in an unstrained dsDNA helix is 10.5. The bottom row shows the outputs of the corresponding CanDo models. These illustrate the approximate shape and local helix mobility.

**Table:** 

Twist Schematic	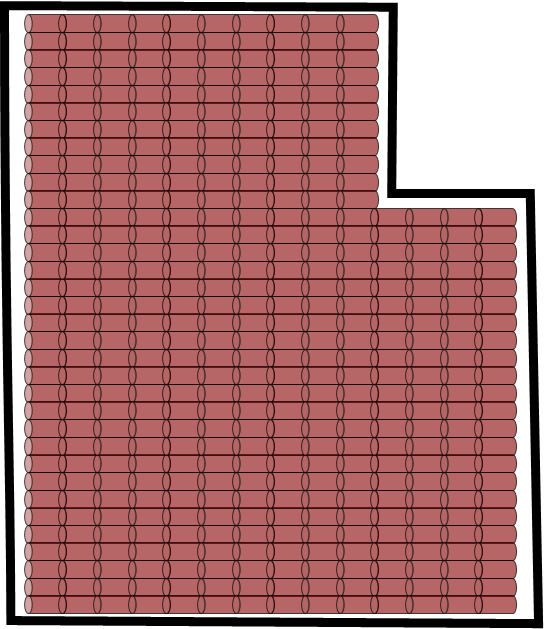	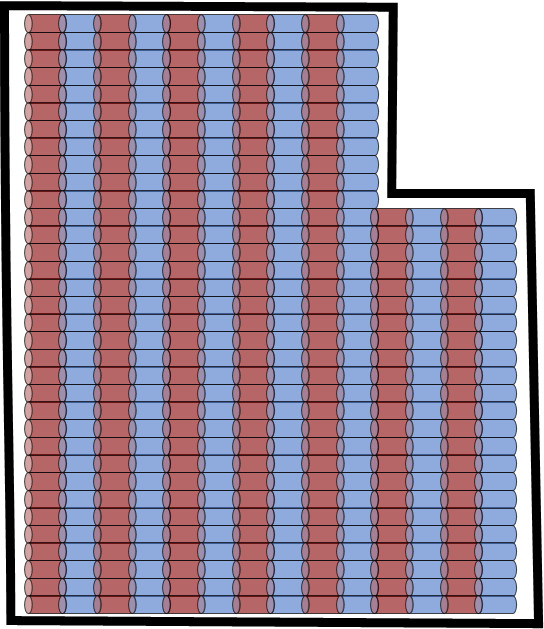	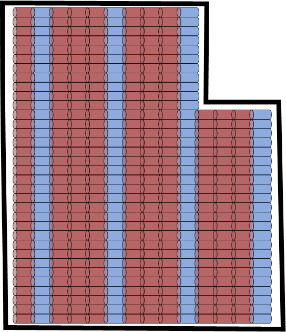	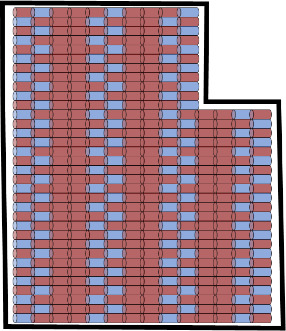
Average Twist Values	Default Length: slightly overwound 10.66 bp/turn 33.75A/bp	Alternating Skips: 10.333 bp/turn 34.84A/bp	A1/4 Skips: 10.5 bp/turn 34.28A/bp	A1/4 Skips staggered: 10.5 bp/turn 34.28A/bp
CanDo Prediction	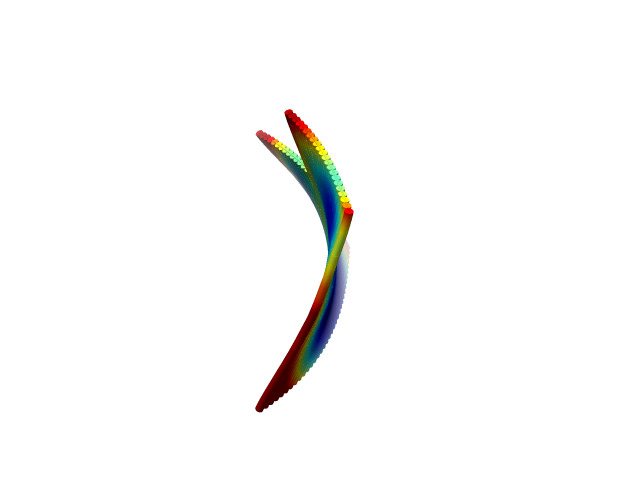	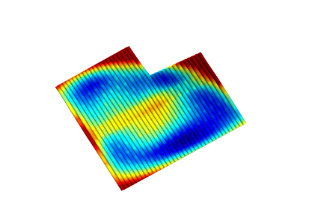	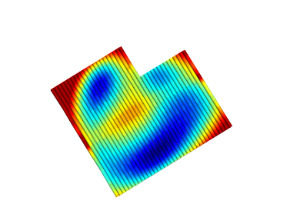	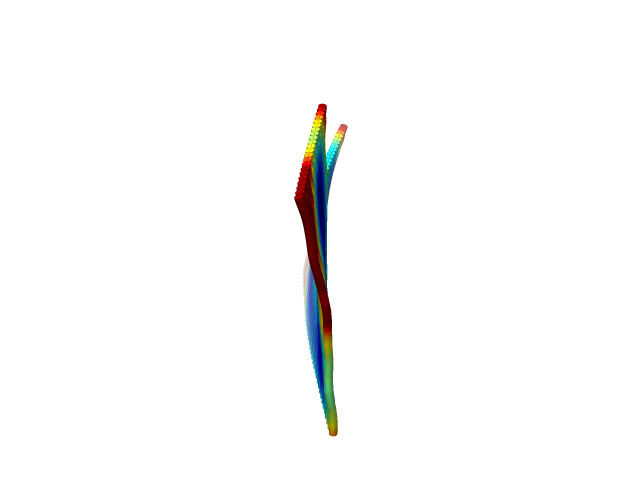

**Fig. 23:**
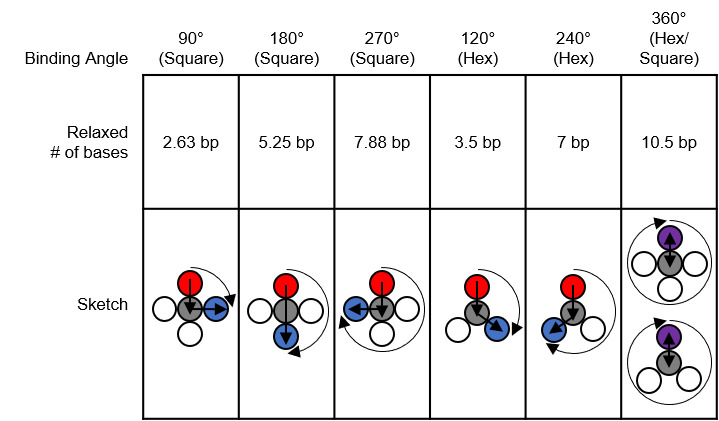
Relaxed number of bases for common helix binding angles. If the distance between two crossovers is greater than a full turn, to determine whether it is over- or underwound, subtract the largest integer multiple of 10.5 bases possible without resulting in a negative number.

*Note: Use of this strain to generate curved surfaces is addressed in Ref.* [49]. 

The "relaxed" number of bases between crossovers depends on the enforced angle between helices, as shown in Fig. 23. While this may be a useful reference, it is a worthwhile exercise to independently derive these numbers. On the hexagonal lattice, the relaxed number is either an integer or ± 0.5 bases. While the square lattice lies flat for easier AFM imaging, compensation of residual strain/torque is much easier for the hexagonal lattice.

The reader is advised to save backup copies of the CAD file between modification attempts, and to regularly use finite element analysis prediction for each step to ensure specifications can be met. Given the number of files generated, a rational file naming and organizing scheme is essential.

## 5 Additional Conceptual Tools

### 5.1 Folding Information and its Representation

A common concern for designers of DNA origami systems is, among the multiplicity of design options, how to determine if one is better or worse than others. Current understanding is insufficient to answer this question, but a few conceptual tools can illustrate how structures are different.

One factor that is known to be important in origami folding are entropic penalties, which occur when the topology of the scaffold is forced to change *via* staple binding. The total entropic cost of transforming the flexible ssDNA scaffold into a single relatively inflexible block is small compared to the energy gains associated with binding. However, this cost is levied unevenly, and it significantly discourages long-distance folds from occurring until other staples reduce that distance. Fold distances may be visualized within the routing pattern, circle plots, histograms, and square plots.

The routing pattern, discussed in the instructions, conveys the desired shape of a structure and is a central focus of the design process.
The circle plot illustrates the initial distance each fold must bridge. In the circle plot, the scaffold is represented by the outside circle, while each arc indicates a fold. One limitation of the circle plot is that the vast majority of folds are too small to see clearly.
Histogram plots of fold distances readily depict small folds and can be used to determine fold-distance statistics, but they contain no information on fold nesting.
The square plots that we have devised, and that we present here for the first time, attempt to combine the most useful aspects of circle plots and fold histograms. The scaffold in the square plot is represented by the *x* axis, and in the case of a circular scaffold, it should be noted that that origin and the furthest extent represent adjacent points on that scaffold. Folds are plotted as lines between their start/stop positions, with the *y*-axis position corresponding to the initial fold distance, equal to the difference between the start and stop positions on the scaffold for that fold. Folds plotted above/below each other will directly reduce the looping entropy penalties for each other. In this way, cones that run from the top to bottom of the square plot indicate folding pathways along which a long stretch of scaffold is "zipped" closed. To emphasize this feature, a transparent shading was applied from each fold down to the bottom of the plot. Darker regions reflect many nested folds on top of one another. In short, darker shading regions imply a folding "pathway" in which some shorter folds must occur first to enable subsequent folds.

### 5.2 Congruent Shapes of Various Scaffold Routing

To illustrate the plots described in Sec. 5.1 and the plethora of design choices, we created four different DNA origami of identical, or nearly identical, 2D footprint. In an AFM or TEM, these structures would be difficult to distinguish from one another.

The two designs most typical of 2D origami to date are the vertical and horizontal designs (Fig. 24). The scaffold routing for these two designs places a seam that bisects the rectangular footprint along a cardinal axis. As the rectangle has a high aspect ratio, this results in very different loop distributions. The vertical origami has a very large number of short-fold-distance nucleating sites, but many more long-distance folds as the system zips up the seam. In contrast, the horizontal origami has relatively few very long or very short folds, and a large number of medium-distance folds.

The other two designs presented here are less typical but represent conceptually simple modifications to the horizontal design. The horizontal interdigitated design moves the scaffold crossovers of the seam back and forth so that they are no longer contiguous, broadening the distribution of fold distances. The woven structure is the result of taking each crossover position and, if possible, having the scaffold jump between helices rather than the staple.

**Fig 25:**
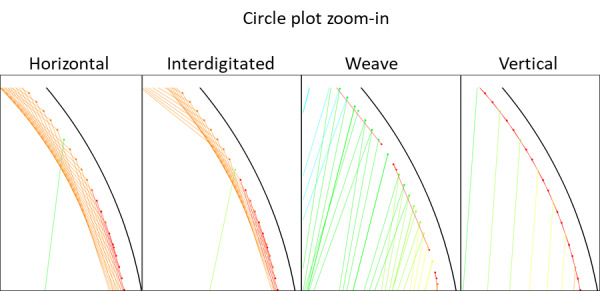
Zoom-in of respective circle plots illustrating the short folds, which can be difficult to see in circle plots.

In evaluating a design, we recommend use of the circle plot to confirm the number and symmetry of substructural units in the origami, as the circle plot can be visualized on "top" of the routing for troubleshooting. A drawback of the circle plot, shown in Fig. 25, is that it poorly depicts the very short folds, which often comprise the majority of a structure. The square plot is more useful for identifying the degree to which folds will occur in parallel or *via* a few path-dependent "zippering" interactions.

The examples in Fig. 24 illustrate design differences in cases where numerous designs occupy the same footprint. Note: The routing patterns, particularly for the vertical origami, are not to scale.

The "horizontal" origami has helices running parallel to its long side and a central seam, and it would be rigid in the long direction but flexible along the short direction. If one only looked at the routing map, it would be easy to believe this origami has the greatest number of long folds because the scaffold has the fewest turns.

The "vertical" origami has helices running parallel to its short side and a long central seam, and it would be flexible in the long direction but rigid in the short direction. Because there are many positions where the scaffold routes back and forth in the short direction, there are large numbers of short folds, shown in the histogram.

The "interdigitated horizontal" origami illustrates how the fold distances in the horizontal origami would change if the seams were shifted/interdigitated. This example shows how simple changes to the routing pattern can dramatically change the distribution of loop distances.

Finally, the "woven" origami takes the horizontal origami design and maximizes the number of positions where the scaffold is jumping between helices, rather than the staples.

While different routing patterns result in quite different fold distance distributions, we do not yet know if those differences have a significant impact on the quality of self-assembly.

**Fig. 24:** Example representation of congruent designs. For each design, Top-Left: scaffold routing, Top-Right: histogram, Bottom-Left: circle plot, Bottom-Right: square plot. **Table:** 

**Horizontal Origami**		
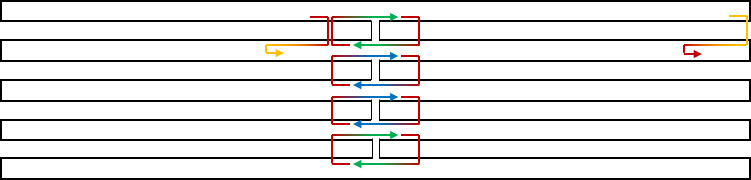		
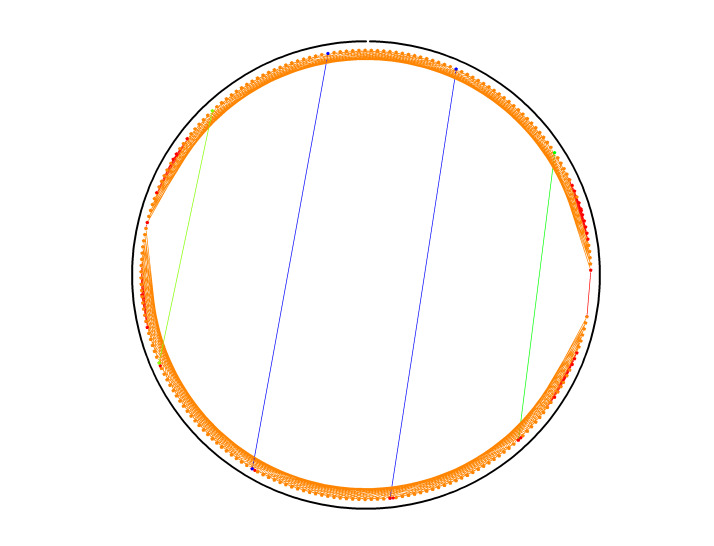		
**Horizontal Origami Interdigitated**		
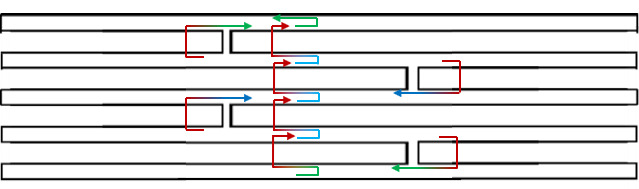		
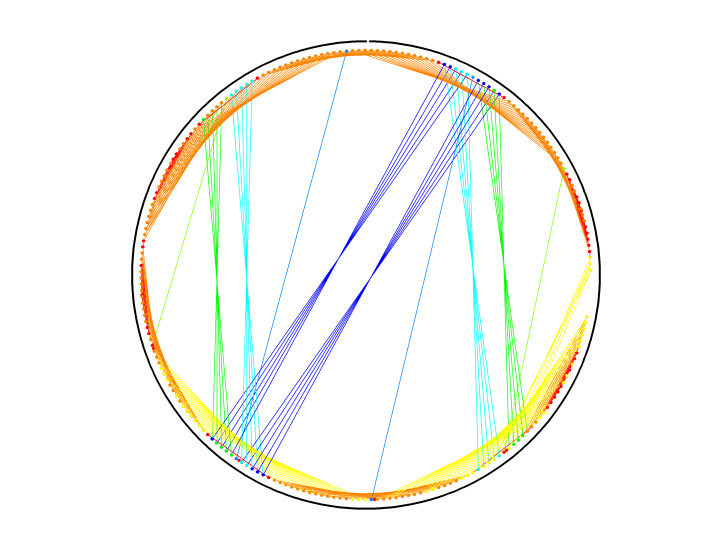

**Fig. 24:** Example representation of congruent designs. For each design, Top-Left: scaffold routing, Top-Right: histogram, Bottom-Left: circle plot, Bottom-Right: square plot (continued). **Table:** 

**Woven Horizontal Origami**		
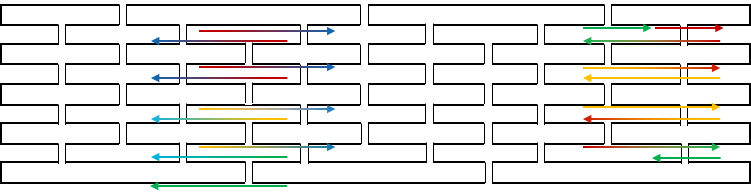		
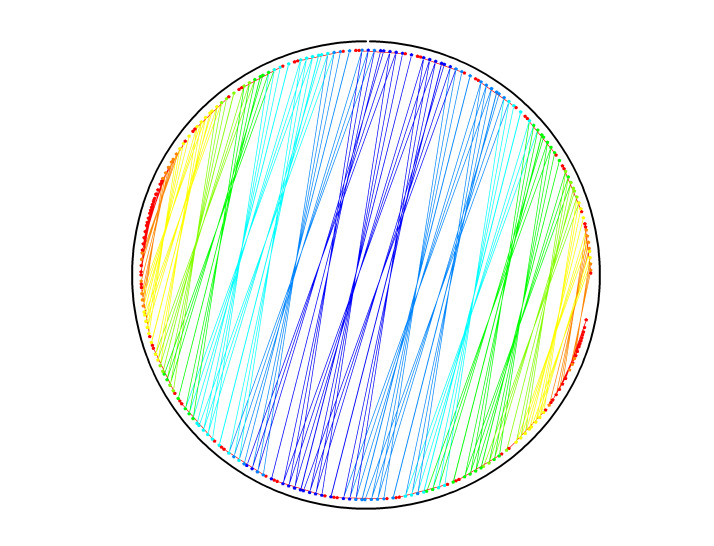		
**Vertical Origami**		
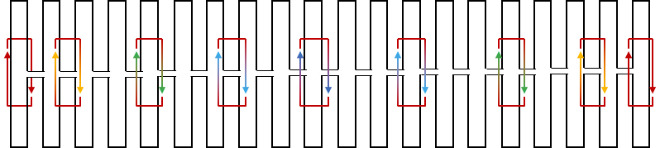		
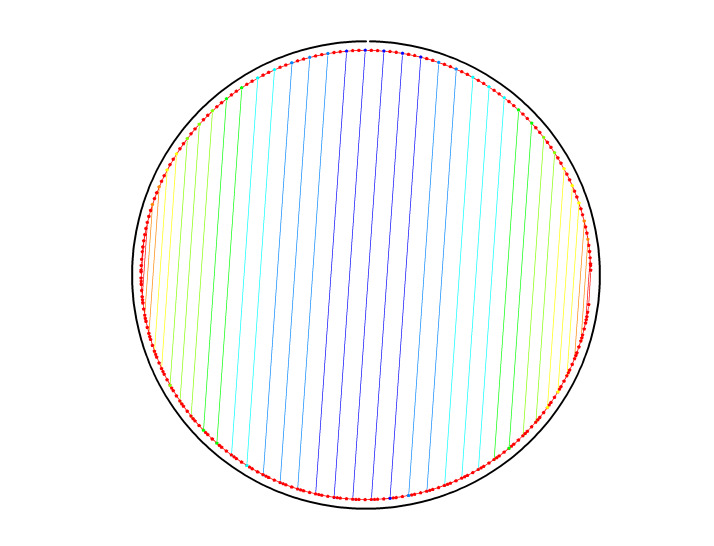

### 5.3 Staple Extensions for Addressing Functional Units

Sticky ends-short lengths of unbound ssDNA-are used to create addressable sites on the origami to which functional units such as nanoparticles or biomolecules may be added. These sticky ends may be incorporated as a hairpin in the center of a staple or as an extension at either end of the staple. In both cases, it is critical to know which direction relative to the bulk of the origami the sticky end will exit.

As briefly mentioned in the introductory material, the extension will exit the helix based on the position of the minor groove at the relevant base position (Fig. 1 and Fig. 2).

**Fig. 26:**
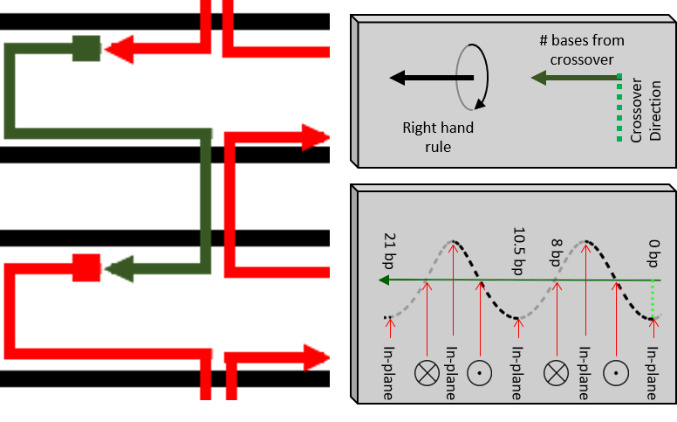
Design example and application of the right-hand rule in placing an addressable sticky end.

This process is illustrated in Fig. 26. The simplest visualization comes from combining the direction of the staple, the direction of the nearest crossover, and the helicity of dsDNA to determine the exit direction for the sticky end *via* the right-hand rule. This can be done by imaging the right-hand-thumb in the 5´→ 3´ direction and the index finger initially in the direction of the crossover. As the hand moves along the dsDNA, the fingers should curl at ≈ 34.3°/base in the 3´ direction. This is approximately 270° per every 8 bases.

One useful exercise is to examine the Rothemund Tall Rectangle [10], which is the basis structure for the notched rectangle shown throughout this text. The Tall Rectangle is often used as a representative 2D structure. Based on its design, all its 5´ and 3´ staple ends exit the origami on the same face. If functional components are needed on both sides of the structure, a minor redesign of the staples is necessary.

Confirming this property, and attempting said staple redesigns, is a worthwhile exercise for designers new to DNA origami.

### 5.4 Programmed and Unintentional Intra-origami Base Stacking

In the introduction, origami were described as being built from dsDNA helices welded together by crossovers. There is an additional critical feature of these helices relevant to DNA origami. While the radial surface of the helix comprises the negatively charged hydrophilic backbone, the end caps comprise the hydrophobic bases. The sides of an origami parallel to the helix direction are hydrophilic, while those perpendicular to the helix direction (with exposed cylinder ends) are hydrophobic.

**Fig. 27:**
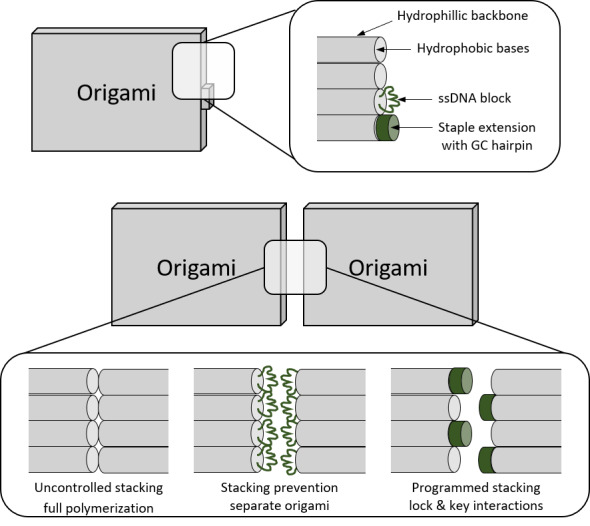
Interorigami base stacking between helix ends, prevention thereof, and programmed base stacking.

As such, the exposed cylinder ends at the edges of DNA nanostructures are prone to reversible, low-energy stacking [12], as shown in Fig. 27.

This stacking can be beneficial, *e.g.*, for surface tiling, or disadvantageous, because it may lead to uncontrolled agglomeration. A tried-and-tested of way to prevent stacking is to place a 4+ base poly-T loop on the staples when they jump between helices at the cylinder ends. If G-C hairpins are placed in these positions on the staples, they can create physical lock-and-key arrangements that only allow stacking between preprogrammed substructures.

### 5.5 Ordering and Naming Conventions

Most DNA nanofabrication CAD tools have a primary export format of comma-separated value, or .csv, files. This is because most oligonucleotide synthesis companies perform bulk orders through .csv workbook files. While the relevant cells from the .csv file can be copied directly into the order forms, it is advisable to take additional time to consider naming conventions.

One DNA origami typically requires 250 staple strands or more, requiring procurement of three 96 deep-well plates, with numerous individual tubes for functionalized staples. Given that one set of plates can last for both a considerable time and for a considerable number of experiments, a consistent naming convention is useful.

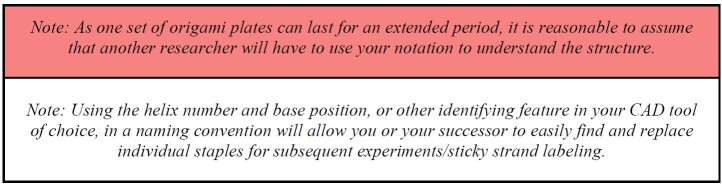



CAD tool .csv export files will typically include information such as the helix number and base position along that helix for each staple. It is advisable to use this information as part of the name in purchasing, to minimize frustration in future attempts to add or remove individual staples.

Toward this end, common text extraction and concatenation tools in workbook programs are useful. Examples include LEFT() and "&" in Excel^®^. Combining these with an autofill function can allow for complex names to be quickly generated for all strands. It is advisable to include properties like a structure name abbreviation, substructure, and helix/base position of the staple.

Default mass ordering spreadsheets will often name the plates "Sheet1" to "SheetN" or "Plate1" to "PlateN." The wise researcher will avoid a freezer full of many structures each with multiple plates labeled with such generic names.

## 6 Summary

By breaking the design process for DNA origami into a sequence of iterative operations involving straightforward decisions and prioritizations, it is possible to streamline the creation of a new design, both minimizing nonproductive tinkering and reducing the likelihood of errors. The general flow of this process is applicable to other types of DNA nanofabrication systems such as single-strand tiles (SSTs) and infinitely tiling systems.

If only one piece of advice from this document is retained by the reader, we strongly suggest that it be the understanding that critical and intentional predesign, including the generation of a complete list of functional requirements, is absolutely necessary to the efficient development of a working structure.
